# Supplementary material for: Modelling Skylarks (Alauda arvensis) to Predict Impacts of Changes in Land Management and Policy: Development and Testing of an Agent-Based Model
Source: PLoS One. 2013 Jun 6;8(6):e65803. doi: 10.1371/journal.pone.0065803 (PMC3675089; doi:10.1371/journal.pone.0065803)
Supplement: Supporting Information S4 — The skylark ODdox as a zipped archive. (ZIP) [file pone.0065803.s004.zip › Skylark_ODdox/globals_0x6c.html]

ALMaSS Skylark ODdox: File Members


ALMaSS Skylark ODdox
 2.0


- Main Page
- Related Pages
- Classes
- Files

- File List
- File Members

- All
- Functions
- Variables
- Typedefs
- Enumerations
- Enumerator
- Macros

- \_
- a
- b
- c
- d
- e
- f
- g
- h
- i
- j
- k
- l
- m
- n
- o
- p
- r
- s
- t
- u
- v
- w
- x

Here is a list of all file members with links to the files they belong to:

### - l -

- l\_cfg\_public\_exit\_on\_set
  : configurator.cpp
- l\_cfg\_public\_warn\_on\_set
  : configurator.cpp
- l\_el\_bug\_glue
  : elements.cpp
- l\_el\_bug\_percentage\_file()
  : elements.h
- l\_el\_bug\_slope
  : elements.cpp
- l\_el\_growth\_daydeg\_magic
  : elements.cpp
- l\_el\_growth\_phase\_shift\_level
  : elements.cpp
- l\_el\_o\_cut\_green
  : elements.cpp
- l\_el\_o\_cut\_height
  : elements.cpp
- l\_el\_o\_cut\_total
  : elements.cpp
- l\_el\_plant\_biomass\_proport
  : elements.cpp
- l\_el\_read\_bug\_percentage\_file()
  : elements.h
- l\_el\_rv\_cut\_aug
  : elements.cpp
- l\_el\_rv\_cut\_green
  : elements.cpp
- l\_el\_rv\_cut\_height
  : elements.cpp
- l\_el\_rv\_cut\_jul
  : elements.cpp
- l\_el\_rv\_cut\_jun
  : elements.cpp
- l\_el\_rv\_cut\_may
  : elements.cpp
- l\_el\_rv\_cut\_oct
  : elements.cpp
- l\_el\_rv\_cut\_sep
  : elements.cpp
- l\_el\_rv\_cut\_total
  : elements.cpp
- l\_el\_rv\_may\_1st
  : elements.cpp
- l\_el\_veg\_heightscale
  : elements.cpp
- l\_el\_veg\_start\_lait
  : elements.cpp
- l\_el\_weed\_glue
  : elements.cpp
- l\_el\_weed\_percent
  : elements.cpp
- l\_el\_weed\_slope
  : elements.cpp
- l\_farm\_cattle\_veg\_reduce
  : farmfuncs.cpp
- l\_farm\_cattle\_veg\_reduce2
  : farmfuncs.cpp
- l\_farm\_event\_do\_it\_prob
  : farmfuncs.cpp
- l\_farm\_herbicide\_kills
  : farmfuncs.cpp
- l\_farm\_insecticide\_kills
  : farmfuncs.cpp
- l\_farm\_pig\_veg\_reduce
  : farmfuncs.cpp
- l\_map\_art\_hb\_core\_thres
  : hedgebanks.cpp
- l\_map\_art\_hb\_nums
  : hedgebanks.cpp
- l\_map\_art\_hb\_seg\_len
  : hedgebanks.cpp
- l\_map\_art\_hb\_tran\_prob
  : hedgebanks.cpp
- l\_map\_art\_hb\_width
  : hedgebanks.cpp
- l\_map\_art\_hedgebanks
  : Landscape.cpp
- l\_map\_chameleon\_replace\_num
  : Landscape.cpp
- l\_map\_check\_polygon\_xref
  : Landscape.cpp
- l\_map\_CIPEmaker\_enable
  : Landscape.cpp
- l\_map\_cropcurves\_file
  : Landscape.cpp
- l\_map\_dump\_enable
  : Landscape.cpp
- l\_map\_dump\_event\_enable
  : Landscape.cpp
- l\_map\_dump\_event\_x1
  : Landscape.cpp
- l\_map\_dump\_event\_x2
  : Landscape.cpp
- l\_map\_dump\_event\_y1
  : Landscape.cpp
- l\_map\_dump\_event\_y2
  : Landscape.cpp
- l\_map\_dump\_exit
  : Landscape.cpp
- l\_map\_dump\_gfx\_enable
  : Landscape.cpp
- l\_map\_dump\_gfx\_file
  : Landscape.cpp
- l\_map\_dump\_map\_file
  : Landscape.cpp
- l\_map\_dump\_margin\_file
  : Landscape.cpp
- l\_map\_dump\_poly\_file
  : Landscape.cpp
- l\_map\_dump\_treatcounts\_enable
  : Landscape.cpp
- l\_map\_dump\_treatcounts\_file
  : Landscape.cpp
- l\_map\_dump\_veg\_enable
  : Landscape.cpp
- l\_map\_dump\_veg\_x
  : Landscape.cpp
- l\_map\_dump\_veg\_y
  : Landscape.cpp
- l\_map\_exit\_on\_zero\_area
  : Landscape.cpp
- l\_map\_farmref\_file
  : farm.cpp
- l\_map\_map\_file
  : Landscape.cpp
- l\_map\_no\_pesticide\_fields
  : Landscape.cpp
- l\_map\_poly\_file
  : Landscape.cpp
- l\_map\_print\_version\_info
  : Landscape.cpp
- l\_map\_read\_farmfile
  : farm.cpp
- l\_map\_renumberpolys
  : Landscape.cpp
- l\_map\_rotation\_files\_prefix
  : croprotation.cpp
- l\_map\_umargin\_width
  : Landscape.cpp
- l\_map\_weather\_file
  : Landscape.cpp
- l\_pest\_ai\_half\_life
  : pesticide.cpp
- l\_pest\_daily\_mort
  : pesticide.cpp
- l\_pest\_diffusion\_grid\_count
  : pesticide.cpp
- l\_pest\_diffusion\_slope
  : pesticide.cpp
- l\_pest\_enable\_pesticide\_engine
  : Landscape.cpp
  , pesticide.cpp
- l\_pest\_insecticide\_amount
  : farmfuncs.cpp
  , pesticide.cpp
  , elements.cpp
- l\_pest\_product\_0\_amount
  : farmfuncs.cpp
  , pesticide.cpp
- l\_pest\_product\_1\_amount
  : farmfuncs.cpp
  , pesticide.cpp
- l\_pest\_productapplic\_enddate
  : farmfuncs.cpp
- l\_pest\_productapplic\_period
  : farmfuncs.cpp
- l\_pest\_productapplic\_startdate
  : farmfuncs.cpp
- l\_pest\_productOrchard\_amount
  : elements.cpp
- l\_pest\_use\_application\_rate
  : pesticide.cpp
- l\_pest\_zero\_threshold
  : pesticide.cpp
- l\_weather\_starting\_year
  : weather.cpp
- last\_treatment
  : treatment.h
- LE\_SIG\_NO\_FUNGICIDE
  : elements.h
- LE\_SIG\_NO\_GROWTH\_REG
  : elements.h
- LE\_SIG\_NO\_HERBICIDE
  : elements.h
- LE\_SIG\_NO\_INSECTICIDE
  : elements.h
- LE\_SIG\_NO\_MOLLUSC
  : elements.h
- LE\_SIG\_NO\_SYNG\_INSECT
  : elements.h
- LE\_Signal
  : elements.h


- Generated on Thu Jan 10 2013 13:15:39 for ALMaSS Skylark ODdox by
   1.8.1.1
